# Evaluation of musculoskeletal workload of manual operating tasks using a hydraulic jack based on ergonomic postural analysis and electromyography: A case study of non-professional young male users

**DOI:** 10.3233/WOR-210079

**Published:** 2022-06-21

**Authors:** Atsushi Sugama, Takahiro Nishimura, Kouki Doi, Shigenobu Shimada, Manabu Chikai, Kiyohiko Nunokawa, Shuichi Ino

**Affiliations:** a Risk Management Research Group, National Institute of Occupational Safety and Health, Japan, Kiyose, Tokyo, Japan; b Department of Teacher Training, National Institute of Special Needs Education, Yokosuka, Kanagawa, Japan; c Faculty of Human Life and Science, Doshisha Women’s College of Liberal Arts, Kamigyo-ku, Kyoto, Japan; d Technology and Management Support Department, Technology Evaluation Support Section, Tokyo Metropolitan Industrial Technology Research Institute, Koto, Tokyo, Japan; e Behavior Information Design Group, Human Informatics and Interaction Research Institute, National Institute of Advanced Industrial Science and Technology, Tsukuba, Ibaraki, Japan; f School of Human and Social Sciences, Tokyo International University, Kawagoe, Saitama, Japan; g Graduate School of Engineering, Osaka University, Suita, Osaka, Japan

**Keywords:** Musculoskeletal loads, hydraulic jack, manual tasks, postural loads, electromyography

## Abstract

**BACKGROUND:**

Manual operations of the hydraulic jack device can become ergonomic stressors for the musculoskeletal system because of the required operational forces, muscle activities, or working postures. However, the usability of the hydraulic jack has not been fully explored for non-professional personnel.

**OBJECTIVES:**

To evaluate the musculoskeletal loads during manual operations of a hydraulic jack based on the ergonomic postural analysis and electromyography

**METHODS:**

Nine men operated the lever of a hydraulic jack with three positions: parallel to and near (P-N), parallel to and far from (P-F), and orthogonal to the jack lever (O). Postural loads were evaluated by Loading on the Upper Body Assessment (LUBA), an ergonomic observational method, and were classified into action categories. The surface electromyogram of eight muscles and the subjective sense of burden were also measured.

**RESULTS:**

The initial force for lever pushing reached 40–80 N and exceeded the recommended forces for the unusual postures. The overall assessment of LUBA showed that 31% of working postures observed in O position require immediate consideration and corrective action and the maximum holding time estimated was < 1 minute. The postural load increased due to the shoulder joint abduction in the P-F and O positions and due to the trunk rotation in O position.

**CONCLUSIONS:**

The results suggest that operating the hydraulic jack cause considerable postural loads and manual forces insufficient for several minutes of manual task. Therefore, improving working methods and tool designs are needed to improve usability and decrease the risk of musculoskeletal disorders during jack operations.

## 1 Introduction

Manual operations of the hydraulic jack device, involving repetitive use of hand tools, awkward postures, or forceful exertions, can become ergonomic stressors for the musculoskeletal system [1]. These stressors increase the risk of musculoskeletal or nervous system disorders [2]. For example, firefighters (FFs) are known to be highly at risk for musculoskeletal injuries and disorders compared with other occupations. The leading cause of injuries of FFs and emergency medical technicians are strains, sprains, and muscular pain from overexertion or falls (39.7%) [1], and these accounted for 59% of all injuries in non-fire sites [3]. This is considered partly due to frequent manual tasks in the workplace [4]. The ergonomic stressor is associated with the poorly ergonomically designed hand tools, tasks, or work environment. Therefore, some studies suggest rescue tool or equipment improvements for professional use, such as transportation equipment [5–7] and respiratory support equipment [8].

However, the usability of the hydraulic jack device for non-professional users has not been fully discussed. The hydraulic jack is a typical manual tool to be used as the repair tool for replacing car tires [9, 10] or the emergency equipment [11, 12] for ensuring an evacuation route and rescuing individuals. The intended users of the hydraulic jack at home and workplaces are not well trained in those operations and do not have enough physical strength compared with professionals such as FFs. A safer kinetic intensity level may be necessary if the musculoskeletal workload is high enough to consider the change of tasks or equipment design. Therefore, examining the effects of ergonomic hazards and minimizing the risk of musculoskeletal disorders during the jack operation are needed.

From an engineering perspective, the physical loading is generated by the product of risk factors of posture, forces, and time and the interactions may sometimes increase the total risk (increased probability and severity of injuries) considerably [13]. Therefore, this study assesses the ergonomics risks for the musculoskeletal workload based on static working postures and manual handlings [14, 15].

For pushing and pulling tasks included in the jack operation, ISO 11228-2 [16] provides the reference criteria based on “Snook Table.” [17] These data indicate that the output level depends on the work duration and task frequency and decreased maximum push/pull forces due to repetitive motions of the upper limb for the jack operation. Therefore, measuring the actual state of force exertion level during the jack operation contributes to a better understanding on the risk of musculoskeletal workload.

Another consideration is static postural workloads due to postural constraints in kneeling position, trunk twisting or tilting, and excessive wrist bending, as described in ISO 11226 [18]. The working posture is generally evaluated based on ergonomic observational techniques, such as OWAS [19] for the entire body, RULA [20] for the upper limb, and LUBA [21] for the upper body. These methods evaluate the angular deviation of a body from the neutral position [22]. To identify and quantify postural stress during work, several methods have been used by visual observation or through the digital human system [23, 24]. Postural loads during squatting and kneeling postures on the lower limbs have been evaluated in several studies [25, 26]. However, the mechanics and kinematics of the upper body motion have not been fully evaluated for the jack operation. Although local muscle exertion and fatigue may affect the usability of manual tools, the strain on local neck, shoulder, and arm muscles caused by the upper limb motion during the jack operation has been controversial.

Under these states, we evaluated postural loads and muscle activity using the LUBA technique and surface electromyography (EMG) under major operating positions relative to lever handles.

## 2 Methods

### 2.1 Participants in the experiment

Nine right-handed men aged 20–27 years participated in this study. All participants were in good health without medical history of musculoskeletal injuries within the last six months. The mean and standard deviations of age, height, and weight were 23.1±2.0 years, 165.9±5.5 cm, and 60.1±5.6 kg, respectively. An oral overview of the experiment was presented to each participant to obtain their informed consent. Additionally, this experiment was conducted based on the “Ethical Guidelines for Research” of the National Institute of Special Needs Education.

### 2.2 Experimental apparatus and procedure

Rescuers from fire departments often use hydraulic jacks to rescue the victim trapped under large objects like vehicles, machinery, and structures. Before the experiment, some rescue teams in the fire department in Japan were interviewed to observe how they handle hydraulic jack devices. The device operator usually kneels on one knee and holds the lever with one hand, watching the lifting part of the jack. Then, they adjust their working posture for task demands depending on the location and situation of worksites. Through this survey, some operators reported that the handling position of the lever largely affects their physical burden. Typically, the body is oriented toward the direction parallel or orthogonal to the lever of the jack. Besides, they prefer to grip the lever as close to their body as possible to reduce the burden; however, they are not often able to approach close to the lever because of the space available on worksites. Therefore, this study controlled the body position relative to the jack lever as an experimental factor.

**Fig. 1:**
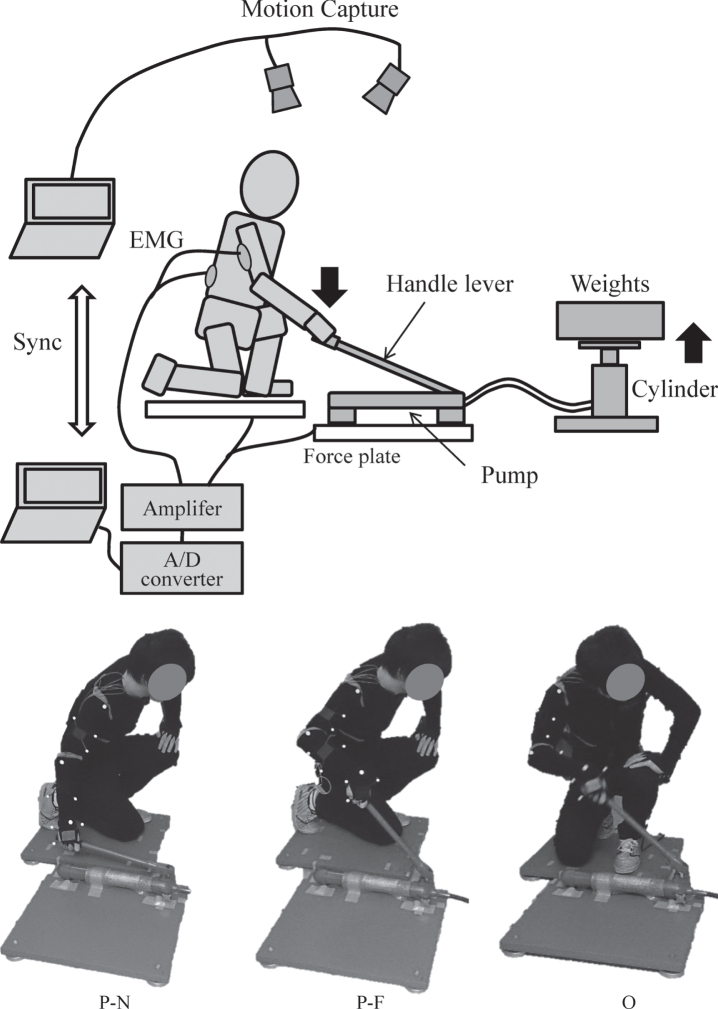
Experimental apparatus and body positions relative to the jack lever. P-N: parallel to and near the lever, P-F: parallel to and far from the lever, and O: orthogonal to the lever.

Experimental conditions were three body positions (Fig. 1): locating parallel to and near the lever (P-N), parallel to and far from the lever (P-F), and orthogonal to the lever (O). The distance between the body and lever was controlled only under the parallel position because participants were having difficulties in reaching the lever from a far position under the orthogonal position. Experimental conditions were completely randomized, and measurements were repeated five times under each condition.

Participants were required to kneel on one knee on two force plates (9286AA; Kistler Instrument Corp., Amherst, NY) and to handle the lever up and down at a constant speed. The operation speed was set at one handle motion (lifting or lowering) per second. The duration of each trial was set as 30 s. During the task, the participant was required to gaze at the lifting part of the hydraulic jack. This study used a hydraulic jack system (Hydraulic unit Porto-Power; Blackhawk Co. Ltd., France) consisting of a pump connected to a cylinder with a hose. As the lifting loads, the 100-kg iron plate weights including the attachment were fixed on the top of the cylinder. The maximum pressure generated by this pump is 70 MPa (700 kg/cm^2^) so that the cylinder used in this study has the maximum operating force of 10 tons. The generated power follows Pascal’s principle using the equation *F* = *P*×*S*, where *F* is the force applied onto the cylinder, *P* is the hydraulic pressure generated by the pump, and *S* is the cross-sectional area of the cylinder.

### 2.3 Measurement and analysis

This study hypothesized that jack operators are exposed to two types of musculoskeletal risk factors: force exertion required for jack handling and holding the static postures during tasks. Therefore, this study evaluated the activity level of muscles using surface electromyogram (sEMG) and the postural loading using a posture assessment technique.

#### 2.3.1 EMG

The activities of eight muscles in the upper limb and body trunk were recorded by sEMG amplifiers (SX230; Biometrics Ltd., UK). The selection of muscles was established based on the prior studies for the manual device operation as a hydraulic pallet jack [27] and a jackhammer [28]. Five muscles were selected as muscles that contribute to the movement on the sagittal plane: the anterior deltoid (AD), posterior deltoid (PD), biceps brachii (BB), triceps brachii (TB), and erector spinae (ES). Three muscles were selected on the frontal plane: the lateral deltoid (LD), sternocostal part of the pectoralis major (SP), and trapezius (TP). Raw signals were recorded at 500 Hz and bandpass-filtered with a 5–200 Hz cutoff frequency. The noise eliminated signals were smoothed using a low-pass filter with a cutoff frequency of 10 Hz.

Before the experiments, the signal during the maximum voluntary contraction (MVC) was recorded based on the manual muscle test for each muscle [29]. Filtered signals were converted to relative MVC values (% MVC) to compare the magnitude among participants. Then, time-averaged values from 0–30 s were calculated.

#### 2.3.2 Postural loads

Postural stress during jack tasks was assessed with Loading on the Upper Body Assessment (LUBA) [21], a postural classification technique that an observer classifies the static working posture into a few categories based on joint angle criteria. A category for each joint motion provides a relative discomfort score. This score is given based on experimental data for the index of perceived discomfort, expressed as numerical ratio scores [21]. The postural load index (PLI) is defined as combined individual scores for joint motions on the neck, shoulders, upper back, lower back, elbows, and wrists/hands. PLI indicates musculoskeletal loading associated with the whole upper body posture. Postures with a PLI of≤5 are classified into action category (AC) 1, indicating acceptable conditions. Postures with PLI of 5–10 are categorized into AC 2, requiring further investigation and corrective changes. For postures with PLI of 10–15 (AC 3), immediate corrective actions are required through redesigning the workplace or working methods. Finally, postures with PLI of > 15 (AC 4) require immediate consideration and corrective action [21].

To conduct the assessment with LUBA, the upper body posture was captured using a motion capture system (OptiTrack; NaturalPoint Inc., United States) with ten infrared cameras (Fig. 1). Reflective markers with 14 mm in diameter were placed on the left and right acromion and hip joints, as well as the upper arm, elbow joint, forearm, and wrist joint of the right arm of participants. Grayscale images of reflective markers were recorded at 100 fps. Coordinates of detected markers were converted into the tilt of body segments and joint angles on the two-dimensional projective planes similarly as the definition of LUBA [21]. The flexion/extension and lateral bending angles of the back (trunk) are calculated based on the line connecting centers of acromia and hip joints. The rotation angle of the back is defined as the angle between the hip joint line and the shoulder line projected on a horizontal plane. Additionally, the upper arm angle is defined as the line between the acromion and elbow, and the forearm is defined as the line connecting the elbow wrist joints. Joint angles averaged for 30 s were used to calculate the relative discomfort score. Then, PLI and AC were calculated for each experiment.

#### 2.3.3 Subjective sense of burden

Participants answered a questionnaire about the burden for their whole-body after each experiment as the subjective sense of burden. The sense was assessed on a scale of one to five: 1 (feel minimal burden), 2 (mild), 3 (moderate), 4 (considerable), and 5 (extreme).

#### 2.3.4 Statistical analysis

Effects of experimental factors on measured indices were compared using the analysis of variance (ANOVA) with a two-way factorial design (body positions and participants) and a post hoc Tukey’s test. Sphericity was evaluated using the Mauchly sphericity test. To evaluate AC, the chi-square test and residual analysis technique were used. The statistical significance level of all tests was set at 5%. Data analyzes were performed using BellCurve for Excel version 3.21 (Social Survey Research Information Co., Ltd., Japan).

## 3 Results

### 3.1 Exerted force and muscle activity

**Fig. 2:**
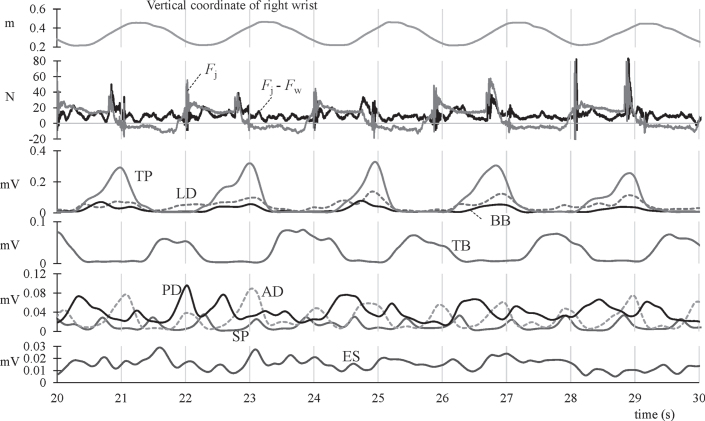
Time waveforms of the vertical coordinate of the right wrist, forces exerted, and sEMG. *F*_J_: the force applied on the jack lever, *F*_W_: the inertial force of body weight, TP: trapezius, LD: lateral deltoid, BB: biceps brachii, TB: triceps brachii, PD: posterior deltoid, AD: anterior deltoid, SP: sternocostal part of the pectoralis major, and ES: erector spinae.

First, we examined the change over time using typical time-series signals. Figure 2 includes the vertical coordinate of the right hand, applied forces on the jack lever, and EMGs during tasks under the P-N position. The vertical coordinate of the right wrist that indicates the movement of the jack lever showed a periodic trend with a period of about 2 s. The force applied on the jack lever *F*_J_ showed a positive value while pushing and negative while pulling. The maximum pushing and pulling forces were about 60 N and 15 N, respectively. Since this signal includes force components of the bodyweight *F*_W_, the delta *F*_J_- *F*_W_ was calculated as the applied force by the upper limb muscle exertions. The magnitude of *F*_J_- *F*_W_ ranged constantly at 10–20 N and momentarily reached 40–80 N. Then, to evaluate functional muscle activities, the eight muscles were classified into four groups based on the timings of activation. In the first group (TP, LD, and BB), the muscles were activated when pulling up the lever, whereas the second group (TB) was activated when pushing down the lever. Muscles in the third group (AD, PD, and SP) were activated twice in a cycle, each for both the pushing and pulling phases. The activation of the last group (ES) remained constant.

**Fig. 3:**
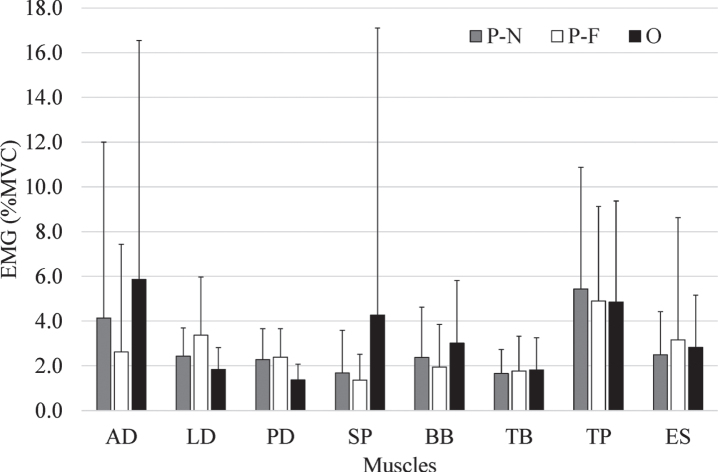
The mean and standard deviation of time-averaged sEMG (% MVC). AD: anterior deltoid, LD: lateral deltoid, PD: posterior deltoid, SP: sternocostal part of the pectoralis major, BB: biceps brachii, TB: triceps brachii, TP: trapezius, and ES: erector spinae.

The activity level of each muscle was evaluated based on relative values (% MVC). Figure 3 shows the mean values of the measured sEMG. The values ranged from 1% –6% of MVC depending on experimental conditions. ANOVA indicated that LD, BB, and PD activities have the main effect on experimental conditions. The activity level of LD in the P-F position was significantly higher than that in other positions. The BB under O position was higher than that of other positions, whereas PD was lower than that of other positions.

### 3.2 Postural load

**Fig. 4:**
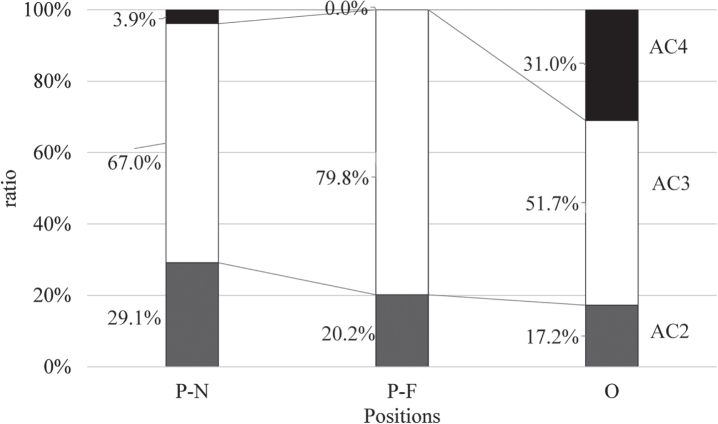
Observed action category (AC) ratios for each position. P-N: parallel to and near the lever, P-F: parallel to and far from the lever, and O: orthogonal to the lever.

Figure 4 shows the AC ratios for each body position. The chi-square test showed significant differences among body positions, and the residual analysis indicated that the AC3 ratio in the P-F and AC4 in O positions were significantly higher than that in other positions. Table 1 lists the mean PLI and relative discomfort scores for joint motions with significant ANOVA differences. PLI did not significantly differ, whereas the largest value was observed in the O position. With regard to relative discomfort scores, back rotation and shoulder flexion/extension in the O position and shoulder adduction/abduction in the P-F position were significantly higher than that in other positions.

**Table 1:** Results of postural load index and relative discomfort scores

Index (mean)	Body position		
	P-N	P-F	O
PLI	9.49	10.03	10.90
Back rotation	0.08	0.23	1.62
Shoulder flexion/extension	0.08	0.00	0.62
Shoulder adduction/abduction	1.23	1.73	0.69

### 3.3 Subjective sense of burden

**Fig. 5:**
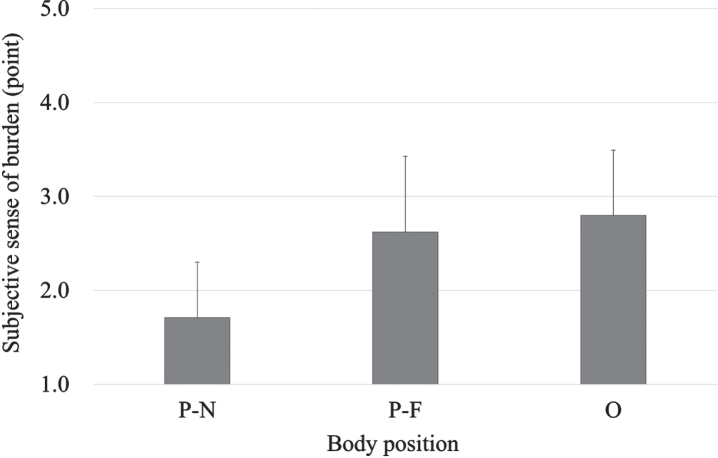
Subjective sense of burden. P-N: parallel to and near the lever, P-F: parallel to and far from the lever, and O: orthogonal to the lever.

Figure 5 shows the mean values of the subjective sense of burden. ANOVA showed the score in the P-N position was lower than that in other positions. Then, relationships among the subjective sense, PLI values, and EMG were investigated using the Pearson’s correlation coefficient. The subjective sense was highly correlated with PLI (*r* = 0.87), but lower with EMG (*r* = 0.44).

## 4 Discussion

### 4.1 Force level

This study investigated the reference level of handling force of the jack lever. First, tasks in this study consist of kneeling on one knee, one hand vertical pushing and pulling of the lever, and the frequency of 30 times per minute. Since the direction of the exerted force and the frequency per minute significantly affect the maximum force output, we refer to the recommended forces for unusual postures by Mital et al. [30], who determined the recommended force for typical cases containing unusual postures such as kneeling, considering the population percentile for both men and women.

The recommended forces for an unusual posture similar to this study, shown as V1 in the guideline, are 9 and 6 kg for male and female industrial workers, respectively [30]. These values were decided according to the intra-abdominal pressure and are similar in all population percentile. Recommended forces for a very high-frequency manual lifting/lowering (22 lifts per min) are 5.2 kg for 90 percentile and 8.6 kg for 50 percentile of male industrial workers, as well as 3.9 kg for 90 percentile and 5.1 kg for 50 percentile for female industrial workers [30]. Based on these references, applied forces to the lever *F*_J_ (60 N for initial pushing and 20 N for sustaining) are considered to be acceptable for male workers but exceed the acceptable values for female workers.

Moreover, to design the handling force for non-professional users, the recommended force should be reduced. For example, in ISO 11228–1 : 2019 [31], the reference mass is determined as 10 kg for the non-occupational use including both 99% of female and male workers, while 23 kg for the occupational use of the adult working population involving 75% and 99% of female and male workers, respectively. For the task requiring similar unusual working postures, a handling force of lighter than 30 N (50% of 60 N for initial pushing) may be suitable for the equipment at home and workplaces.

### 4.2 Postural constraints, EMG, and postural loads

Then, postural constraints on working postures were investigated. First, in the P-N position, the participant was required to place the body close to the lever and lift it vertically in front of the body. This motion was achieved with a combination of the shoulder and elbow joint flexion/extension. In the P-F position, shoulder joint adduction/abduction was also observed because of the lengthened distance to the lever. In the O position, the upper body was rotated to gaze at the moving part of the jack. The rotation consisted of a combination of the rotated pelvis, upper and lower parts of the trunk, and neck. Additionally, the body position was farther from the jack lever than P-N and P-F positions because of the interference of the upper limb with lower limbs. As a result, the participant inclined the trunk forward in order not to flex the upper limb in the O position. This posture is considered to minimize the excessive flexion of the shoulder joint and the moment arm from the shoulder joint to the point of hand force [5].

AD and SP muscle activities significantly in-creased in the O position. Other muscles have no significant differences depending on operating positions. Muscle activity increases in the O position are attributable to the flexion and adduction of the shoulder joint. Conversely, activity levels were up to approximately 5% of MVC in all conditions. Van Dieën and Vrielink [32] showed the relationship between the muscle relative force (% MVC) and endurance time. The endurance time estimated for 4% MVC relative force was 16.5 min for an average and 9.3 min for a minimum. Therefore, the operation of the jack lever was considered to be sustainable, and the local muscle load and fatigue are acceptable.

Postural loads assessed by LUBA ranged from AC2 to AC4. Kamalinia et al. [33] analyzed PLI values and ACs in assembly workers using LUBA. The AC ratio for all workers consisted of 5.7% of AC1, 79.8% of AC2, and 14.5% of AC3, with the mean PLI of 7.7±1.8. These indicate that PLI and ACs in this study were considerably higher than the usual professional works, and tasks with the hydraulic jack require considerable postural loads. The maximum holding time (MHL) was also estimated by Kee and Karwowski [21] based on PLI using a linear equation (MHT = –0.89 ^*^ PLI + 14.80). Based on this equation, MHL for 10 points of PLI is 5.9 min. Estimated MHLs for tasks in this study ranged from 5.1 (O position) to 6.35 (P-N position) min. The endurance time estimated from the relative muscle force and the MHL estimated from PLI suggest tasks in this study are acceptable to continue for approximately 5 min.

### 4.3 Comprehensive assessment of workload during the hydraulic jack operation

Finally, we assess the workload during the hy-draulic jack operation based on manual forces, local muscle activities, and postural loads. The primary workload was to maintain the static working posture and the required initial force for the lever operation, whereas local muscle activities and endurance time are acceptable. These results are supported by relatively higher correlations between the subjective sense of burden and LUBA values.

Since the change of the body position could not reduce the workload under the acceptable level, inherent improvement of device design is required. For example, actuators with lighter handling force are needed for repetitive physical exertions to reduce the manual force. The base structure of the jack may contribute both less postural loads and greater force exertions because the hydraulic jacks set on higher places help exert greater manual forces compared to those set on the ground. Besides, improving the tilt of the lever handle may contribute to reduce postural loads, decrease the elevation and abduction of the shoulder joint. After these inherent improvements against ergonomic hazards, disseminating the proper way to use for non-professional persons is needed to prevent high-risk postures on sites.

The knowledge obtained in the present study contributes to publishing the documents or guidelines [12] for non-professional jack users so that they can prepare the emergency equipment at home and at the workplace and ensure to have the right knowledge on appropriate actions. Besides, for a document for professions, the US Fire Administration shows ergonomic principles for hand tool use including hydraulic lifts [1]. Referring to these principles, focusing on the repetitive use of hand tools, awkward hand positions, and forceful exertions, are also preferable for non-professional users.

### 4.4 Limitation

Although the present study targeted the young male participants, personal factors such as age and gender are needed to be examined in the future study. The older or female participants are considered to have lower average values of maximum force and wider variation within the groups. These musculoskeletal characteristics decrease the recommended forces and need to consider the design of the required force depending on the composition of a targeted group.

During the actual tasks, hydraulic jacks may be operated at a faster speed. Then, the muscle activities due to dynamic motion would increase and may act as the dominant part for the sense of burden. To examine the effect, further study controlling the operating speed is needed. For a better understanding of the relationship between the dynamic operation of the jack system and muscle activities, multivariable analysis dealing with the working posture and EMG as variables might be effective. Some studies investigated static physical tasks introducing the comprehensive evaluation function by the sum of multiple EMG values [34], minimizing the square sum of the ratio of exerted tension of each muscle to maximum muscular tension [35], solving a minimization problem of a cube for each muscular tension divided by the muscle cross-sectional area [36].

## 5 Conclusions

This study evaluated the workload on the upper body during hydraulic jack operations based on the ergonomic postural analysis, muscle activities, exerted manual forces, and subjective sense of burden. Results showed that > 60% of postures observed required corrective actions immediately through redesigning the workplace or working methods. Particularly, in a kneeling position orthogonal to the lever, > 30% are determined as the worst category that requires immediate consideration and corrective action. These postural loads increased depending on the shoulder joint abduction when the operator locates far from and parallel to the lever, while that increased due to trunk rotation and shoulder joint flexion when positioning orthogonal to the lever. Besides, the initial manual force should be decreased for non-professional persons. Improving these awkward positions and the required force during the jack operation decreases the ergonomic risks and extends the maximum holding time for rescue activities.
